# Context and Barriers to the Prescription of Nonoccupational Postexposure Prophylaxis Among HIV Medical Care Providers: National Internet-Based Observational Study in China

**DOI:** 10.2196/24234

**Published:** 2021-03-11

**Authors:** Haibo Ding, Zehao Ye, Weiming Tang, Xiaojie Huang, Hui Wang, Sitong Cui, Yongjun Jiang, Wenqing Geng, Junjie Xu, Hong Shang

**Affiliations:** 1 National Health Commission Key Laboratory of AIDS Immunology (China Medical University) National Clinical Research Center for Laboratory Medicine The First Affiliated Hospital of China Medical University Shenyang, Liaoning Province China; 2 Key Laboratory of AIDS Immunology Chinese Academy of Medical Sciences Shenyang China; 3 Key Laboratory of AIDS Immunology of Liaoning Province Shenyang China; 4 Collaborative Innovation Center for Diagnosis and Treatment of Infectious Diseases Hangzhou China; 5 Project-China University of North Carolina at Chapel Hill Guangzhou China; 6 Center for Infectious Diseases Beijing You'an Hospital Capital Medical University Beijing China; 7 Shenzhen Third People’s Hospital Shenzhen China

**Keywords:** WeChat, nonoccupational postexposure prophylaxis (nPEP), HIV medical care providers, training, key populations, internet, China, social media, barrier, prophylaxis

## Abstract

**Background:**

Nonoccupational postexposure prophylaxis (nPEP) is an effective HIV biomedical prevention strategy. The research and use of nPEP are mainly concentrated in the developed world, while little is known about the knowledge, attitudes, and practices of nPEP among HIV medical care providers in developing countries.

**Objective:**

We aimed to assess the nPEP knowledge and prescribing practice among HIV medical care providers in mainland China.

**Methods:**

HIV medical care providers were recruited in China during May and June 2019 through an online survey regarding nPEP-related knowledge, attitudes, and clinical prescription experiences. Multivariable logistic regression was performed to identify factors associated with prescribing nPEP among HIV medical care providers.

**Results:**

A total of 777 eligible participants participated in this study from 133 cities in 31 provinces in China. Of the participants, 60.2% (468/777) were unfamiliar with nPEP and only 53.3% (414/777) of participants ever prescribed nPEP. HIV care providers who worked in a specialized infectious disease hospital (vs general hospital, adjusted odds ratio [aOR] 2.49; 95% CI 1.85-3.37), had practiced for 6-10 years (vs 5 or fewer years, aOR 3.28; 95% CI 2.23-4.80), had practiced for 11 years or more (vs 5 or fewer years, aOR 3.75; 95% CI 2.59-5.45), and had previously prescribed occupational PEP (oPEP, aOR 4.90; 95% CI 3.29-7.29) had a significantly positive association with prescribing nPEP. However, unfamiliarity with nPEP (aOR 0.08; 95% CI 0.05-0.11), believing nPEP may promote HIV high-risk behavior (aOR 0.53; 95% CI 0.36-0.77) or result in HIV drug resistance (aOR 0.53; 95% CI 0.36-0.77) among key populations, and self-reported having no written oPEP guideline in place (aOR 0.53; 95% CI 0.35-0.79) were negatively associated with nPEP prescription behavior.

**Conclusions:**

HIV medical care providers have insufficient nPEP knowledge and an inadequate proportion of prescribing, which may impede the scale-up of nPEP services to curb HIV acquisition. The implementation of tailored nPEP training or retraining to HIV medical care providers would improve this situation.

## Introduction

### HIV Epidemic in Key Populations

The Joint United Nations Program on HIV/AIDS (UNAIDS) and World Health Organization (WHO) estimate that 38 million people were living with HIV in 2019, with over two-thirds concentrated in low-income developing countries [1,2]. The epidemic of HIV is concentrated in key populations [3], including men who have sex with men (MSM) [4,5]. There has been an increasing number of new HIV infections in China over the past 5 years [6], with approximately 958,000 people reported living with HIV in 2019 [7]. Data based on the HIV Sentinel Surveillance System in China showed that MSM had an HIV infection prevalence rate of 6.9% in 2018 [8].

### Effectiveness of Nonoccupational Postexposure Prophylaxis

Nonoccupational postexposure prophylaxis (nPEP) is an effective and cost-effective HIV biomedical prevention strategy [9,10]. There have been no randomized controlled trials for nPEP due to ethical considerations, but a case-control study of occupational postexposure prophylaxis (oPEP) demonstrated an 81% reduction in the odds of HIV transmission [11]. nPEP guidelines have been in use by WHO, European AIDS Clinical Society, United States, and Canada for years to offer guidance on nPEP uptake [12-16], and the research and use on nPEP in the developed world is extensive. However, nPEP services are not widely used in most developing countries with relatively severe HIV epidemics, even though some have released their own guidelines. Additional efforts are needed to target nPEP uptake to end the AIDS epidemic by 2030.

### Previous Studies and Existing Gap

HIV medical care providers play an indispensable role in nPEP uptake, especially medication prescription [17]. Previous surveys have reported on HIV care providers prescribing nPEP in developed countries [18-23], most often including factors such as practice specialty, the number of persons living with HIV in treatment, provider familiarity with nPEP, and the nPEP guideline in place [18,19,23]. As these surveys were conducted in developed countries with nPEP guidelines, it is uncertain whether the situation is similar in developing countries without nPEP guidelines. A clear understanding of obstacles encountered by providers in developing countries without nPEP guidelines will be beneficial to the scale-up of nPEP uptake and control of the HIV epidemic.

A positive attitude has emerged recently in China on the use of nPEP for HIV prevention. The Chinese Center for Disease Control and Prevention (China CDC) carried out a pilot program of nPEP among MSM in 7 provinces to promote the uptake of PEP and preexposure prophylaxis (PrEP) between 2018 and 2019 [24]. In addition, China released the Program to Reduce AIDS (2019-2020) to ensure that the HIV epidemic was controlled at a low level, which encouraged the application of nPEP programs [25]. Considering an increasing body of evidence, China released the nPEP guideline in October 2020 [26]; however, little is known about the knowledge, attitude, and practice of nPEP in HIV medical care providers in China. It is necessary to understand the nPEP perception among HIV medical care providers and barriers associated with prescribing nPEP to provide targeted interventions.

### Objectives

We sought to understand nPEP perceptions and practice among HIV medical care providers and factors correlated with nPEP prescription under the current efforts of scale-up of nPEP services.

## Methods

### Study Design and Participant Enrollment

We conducted a nationwide online survey among HIV medical care providers during May and June 2019. After a presurvey to adjust the questionnaire items, a survey invitation was sent to 937 HIV medical care providers from two WeChat groups, “National clinicians group majors in HIV/AIDS” and “National physician platform for communicating of difficult cases in HIV/AIDS.” These WeChat groups are currently the leading online WeChat-based communication platforms for HIV-related clinicians in China, with the largest number of registered HIV-related clinicians. The investigator released recruitment information via the WeChat groups, including the study aims, procedure, and requirements of the survey. Eligible participants completed an anonymous online survey by scanning the QR (quick response) code link of the online questionnaire. Inclusion criteria included being age 18 years or older, self-reported practicing in HIV-related medical institutions, having treated at least one person living with HIV over the past year, and providing online informed consent to the study content and protocol. Each individual was allowed to access the online survey once. Each internet protocol address is restricted to answer only one questionnaire. A 30-yuan honorarium (approximately US $4.50) was paid to each participant through WeChat accounts after completion of the 5 to 10 minute questionnaire survey. We used contact information only for releasing rewards and did not disclose it to others.

### Data Collection

After providing informed consent, participants completed anonymous online questionnaires on sociodemographic characteristics (age, sex, ethnicity, and educational background), hospital types, technical titles, practice specialty, length of practice, nPEP-related knowledge, attitudes, and clinical prescription experiences (Multimedia Appendix 1). The 3 questions on nPEP-related knowledge (with possible answers yes, no, and I don’t know) were as follows: Do you think China has issued national clinical guidelines on nPEP? Do you think unprotected anal intercourse (UAI) risk exceeds percutaneous occupational exposure risk? Do you think percutaneous occupational exposure risk exceeds unprotected vaginal intercourse (UVI) exposure risk?

Data on nPEP-related attitudes (with possible answers agree, neutral, and disagree) were also collected as follows: Do you agree that clinicians have enough time to prescribe nPEP? Do you agree that prescribing nPEP in clinical settings is feasible? Do you agree that prescribing nPEP will promote HIV drug resistance? Do you agree that prescribing nPEP will promote high-risk behaviors?

Additionally, we collected nPEP-related experiences, including the experience of encountering key populations seeking nPEP help and nPEP prescribing history. Before submission, participants could review all items of the questionnaire and make sure mandatory items were completed. To evaluate the impact of geographic HIV epidemic level on prescribing nPEP, we categorized regions into high, middle, and low epidemic levels according to the number of HIV/AIDS cases reported in 2017 (Multimedia Appendix 2). The top one-third of regions were classified as having a high epidemic level, while the bottom one-third were classified as having a low epidemic level. Further, to evaluate the impact of the nPEP pilot program recently conducted by China CDC, we divided the provinces into 2 categories, nPEP and non-nPEP pilot provinces. The study protocol was reviewed and approved by the institutional review board committee of the First Affiliated Hospital of China Medical University ([2019]2015-138-9). We have completed the Checklist for Reporting Results of Internet E-Surveys (CHERRIES) for this study (Multimedia Appendix 3).

### Sample Size Calculation

We calculated the sample size of participants based on the formula of a 2-sided confidence interval for one proportion: N = Z^2^_1−α/2_ × P × (1 – P)/D^2^. For a conservative estimate of sample size, the proportion of nPEP prescription (P) was set to be 0.5. At a 5% significance level (α) and 5% margin of error (D), the smallest sample size was calculated as 384 observations.

### Data Analysis

Category variables were described by frequency and percentage and continuous variables by mean and standard deviation or median and interquartile range (IQR). All the core variables involved in the questionnaire are required. For variables with a missing ratio of less than 5%, we imputed related missing values in the database by mean for continuous variables and mode for categorical variables in the course of data processing. Variables with more than 5% missing ratio would have been deleted, but there were none in this study. For the needs of analysis, we transformed some variables (eg, familiarity of nPEP) into the binary forms yes (extremely familiar, very familiar) or no (generally familiar, not familiar very much, not familiar at all). We used univariable logistic regression to calculate odds ratios (OR) and their 95% confidence intervals for factors associated with prescribing nPEP among HIV medical care providers. Multivariable logistic regression was applied to estimate associations between predictors and nPEP prescribing history after adjustment for age, sex, ethnicity, and educational background. We used SPSS Statistics version 26.0 (IBM Corporation) for analysis. Variables with 2-tailed *P*<.05 were considered statistically significant.

## Results

### Demographic Characteristics

**Figure 1:**
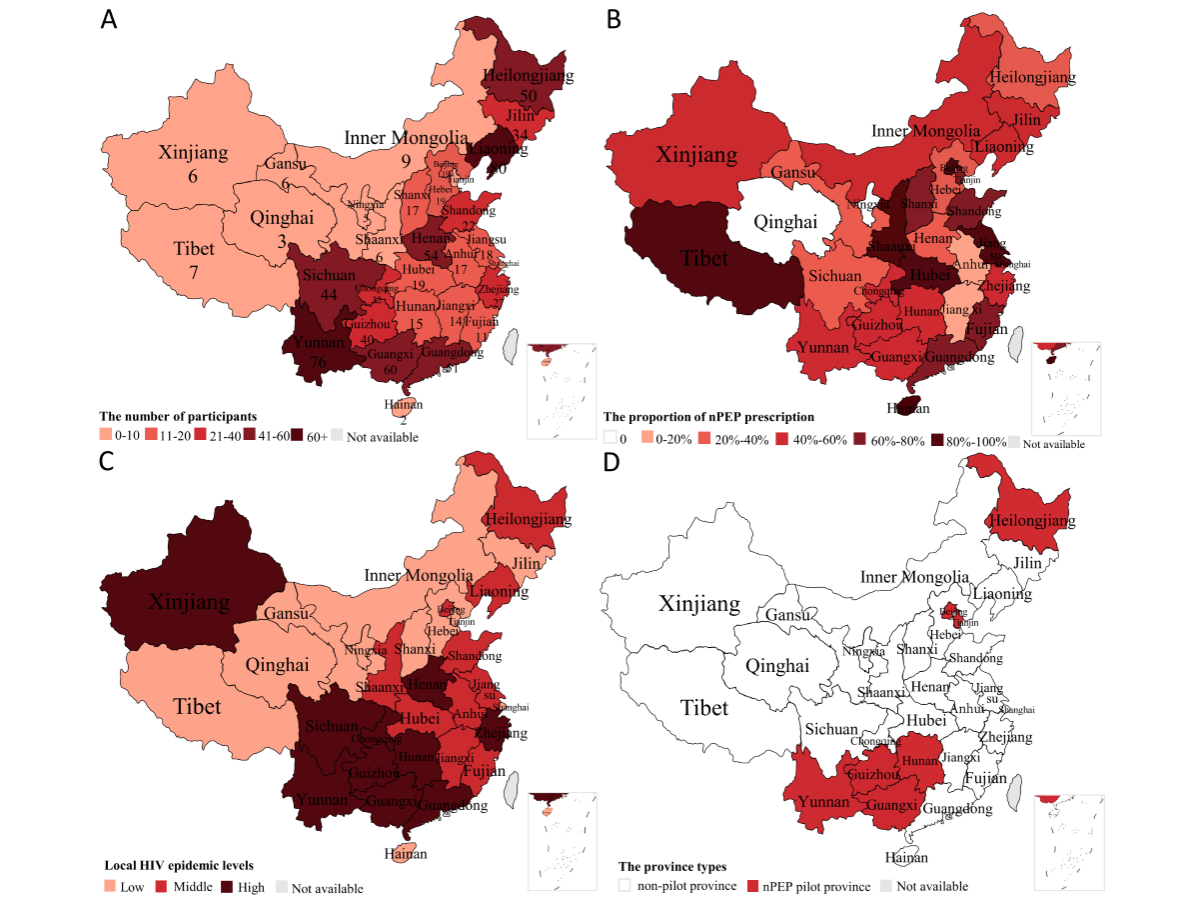
Distribution of participants (A), proportion of nonoccupational postexposure prophylaxis (nPEP) prescriptions (B), HIV epidemic level (C), and nPEP pilot versus nonpilot provinces of 31 total in China (D).

Of the HIV medical care providers reached, 82.9% (777/937) of eligible participants from 133 cities in the 31 provinces of China participated in this study (Figure 1A). Participants had a median age of 42 (IQR 36-48) years. A majority of participants were female (417/777, 53.7%), of Han ethnicity (712/777, 91.6%), had undergraduate or above level of education (743/777, 95.6%), had been in practice for more than 5 years (432/777, 55.6%), and had a technical title of attending physician or above (695/777, 89.4%). Approximately half (394/777, 50.7%) of participants worked at specialized hospitals for infectious diseases (Table 1).

**Table 1:** Demographics and knowledge of HIV medical care providers in China (n=777).

Variable		Total, n (%)
**Age in years**		
	≤25	3 (0.4)
	26-39	302 (38.9)
	40-49	331 (42.6)
	50-59	131 (16.9)
	≥60	10 (1.3)
**Ethnicity**		
	Han	712 (91.6)
	Non-Han	65 (8.4)
**Sex**		
	Male	360 (46.3)
	Female	417 (53.7)
**Educational background**		
	High school/technical secondary school	5 (0.6)
	Junior college	29 (3.7)
	Undergraduate or above	743 (95.6)
**Administrative regions of China**		
	North	71 (9.1)
	Northeast	164 (21.1)
	East	116 (14.9)
	South Central	201 (25.9)
	Southwest	199 (25.6)
	Northwest	26 (3.3)
**nPEP^a^ pilot program province^b^**		
	Yes	267 (34.4)
	No	510 (65.6)
**Local HIV epidemic level^c^**		
	Low	116 (14.9)
	Middle	256 (32.9)
	High	405 (52.1)
**Hospital type**		
	Specialized hospital for infectious diseases	394 (50.7)
	General hospital	383 (49.3)
**Technical title**		
	General physician	82 (10.6)
	Attending physician	274 (35.3)
	Associate chief physician	221 (28.4)
	Chief physician	200 (25.7)
**Clinical practice specialty**		
	HIV care professional	649 (83.5)
	Non-HIV care professional	128 (16.5)
**Practice time in years**		
	≤5	345 (44.4)
	6-10	184 (23.7)
	≥11	248 (31.9)
**Unfamiliar with oPEP^d^**		
	Yes	292 (37.6)
	No	485 (62.4)
**Unfamiliar with nPEP**		
	Yes	468 (60.2)
	No	309 (39.8)
**Do you think China has issued a national clinical guideline on nPEP?**		
	Yes	551 (70.9)
	No	97 (12.5)
	I don’t know	129 (16.6)
**Do you think UAI^e^ exposure risk between males exceeds percutaneous occupational exposure risk?**		
	Yes	667 (85.8)
	No	61 (7.9)
	I don’t know	49 (6.3)
**Do you think percutaneous occupational exposure risk exceeds UVI^f^ exposure risk?**		
	Yes	532 (68.5)
	No	199 (25.6)
	I don’t know	46 (5.9)

^a^nPEP: nonoccupational postexposure prophylaxis.

^b^nPEP pilot programs were conducted by China CDC in provinces Yunnan, Beijing, Tianjin, Heilongjiang, Hunan, Guangxi, and Guizhou.

^c^Local HIV epidemic level was categorized according to the number of HIV/AIDS cases reported in 2017.

^d^oPEP: occupational postexposure prophylaxis.

^e^UAI: unprotected anal intercourse.

^f^UVI: unprotected vaginal intercourse.

### Knowledge, Experiences, and Attitudes

Overall, only 39.8% (309/777) of participants reported that they were familiar with nPEP, and just 6.8% (53/777) correctly answered all 3 nPEP knowledge-related questions (Table 1). Further, 59.3% (461/777) of participants had provided medical services to fewer than 50 persons living with HIV over the past month, 40.2% (312/777) reported that they had encountered key populations seeking nPEP prescriptions over the past 6 months, and 74.0% (575/777) reported that they had a written oPEP guideline in place (Table 2). Among providers from Northwest China, 69.2% (18/26) were unfamiliar with nPEP and only 23.1% (6/26) had provided HIV care to more than 50 persons living with HIV over the past month.

***A survey of participant opinions on the most suitable population for nPEP prescriptions showed that most participants were inclined to prescribe nPEP to people having a partner living with HIV (543/777, 69.9%), people who had been sexually assaulted (485/777, 62.4%), as well as those with histories of sexually transmitted disease (483/777, 62.2%), unprotected sexual intercourse (466/777, 60.0%), irregular visits to the clinic (454/777, 58.4%), drug injection (452/777, 58.2%), and poor drug adherence (402/777, 51.8%). Moreover, 58.9% (458/777) agreed that they had adequate time to prescribe nPEP, and 27.3% (212/777) and 32.9% (256/777) reported that nPEP may promote HIV drug resistance and HIV high-risk behavior, respectively, among key populations. The problems that primarily concerned participants were high drug cost (452/777, 58.2%) and adverse effects of nPEP (438/777, 56.4%; Table 2).

**Table 2:** Experiences and attitudes among HIV medical care providers in China (n=777).

Variable				Total, n (%)
**Number of persons living with HIV in treatment in the past month**				
	≤50		461 (59.3)	
	>50		316 (40.7)	
Ever prescribed nPEP^a^				414 (53.3)
Ever prescribed oPEP^b^				197 (25.4)
Self-reported having a written oPEP guideline in place				575 (74.0)
**Key populations seeking nPEP help** **over** **the past 6 months**				
	Often/occasionally (more than 1 per month)		312 (40.2)	
	Never/rarely (less than 1 per month)		465 (59.8)	
Having barriers for prescribing nPEP in place				236 (30.4)
**nPEP-related attitudes**				
	**Have adequate time to prescribe nPEP**			
		Agree	458 (58.9)	
		Neutral	235 (30.2)	
		Disagree	84 (10.8)	
	**nPEP will promote HIV drug resistance**			
		Agree	212 (27.3)	
		Neutral	308 (39.6)	
		Disagree	257 (33.1)	
	**nPEP will promote HIV risky behavior**			
		Agree	256 (32.9)	
		Neutral	302 (38.9)	
		Disagree	219 (28.2)	
	**Feasible to provide nPEP in place**			
		Agree	712 (91.6)	
		Neutral	57 (7.3)	
		Disagree	8 (1.0)	
	Worry about being blamed for prescribing nPEP due to no nPEP drug indication		583 (75.0)	
	Necessary to have expert consensus for nPEP		693 (89.2)	
	Necessary to establish outpatient for nPEP		620 (79.8)	
	**Concerns about prescribing nPEP**			
		Increased risk behavior	291 (37.5)	
		Poor medication adherence	310 (39.9)	
		HIV drug resistance	238 (30.6)	
		Side effects	438 (56.4)	
		High cost	452 (58.2)	
		No specific guidance for nPEP	159 (20.5)	
		No nPEP drug indication	126 (16.2)	
		Resources reduced for HIV-positive patients	121 (15.6)	
		Other problems	40 (5.1)	

^a^nPEP: nonoccupational postexposure prophylaxis.

^b^oPEP: occupational postexposure prophylaxis.

### Factors Associated With Prescribing nPEP

In total, 53.3% (414/777) of participants had previously prescribed nPEP, among which 38.9% (161/414) reported that they had experienced barriers during the process (Table 2). The proportion prescribing nPEP in each province ranged from 30% to 80% (Figure 1B; Multimedia Appendix 4). The proportions of participants having a history of nPEP prescription were 51.9% (60/414), 56.3% (144/414), and 51.7% (210/414) in provinces with high, middle, and low HIV epidemic levels, respectively (Figure 1C; Table 3), while in the 7 nPEP pilot provinces, 56.2% (150/267) of participants had a history of nPEP prescription (Figure 1D).

Table 3 presents the results of univariable and multivariable logistic regression analyses of factors associated with prescribing nPEP among HIV medical care providers. The forest plot of the results of multivariable logistic regression analysis can be found in Multimedia Appendix 5. After initial adjustment for age, sex, ethnicity, and educational background, we found providers from Northwest regions had a significantly lower proportion of nPEP prescription (vs North China; adjusted odds ratio [aOR] 0.35; 95% CI 0.14-0.89). We further adjusted age, sex, ethnicity, educational background, and administrative regions covariates, and independent factors positively associated with prescribing nPEP were as follows: practicing in a specialized infectious disease hospital (vs general hospital, aOR 2.49; 95% CI 1.85-3.37), working professionally in HIV care (vs nonprofessional in HIV care, aOR 6.13; 95% CI 3.83-9.81), having a technical title of chief physician (vs general physician, aOR 2.16; 95% CI 1.15-4.05), having 6 to 10 years of practice (vs 5 or fewer years, aOR 3.28; 95% CI 2.23-4.80), having 11 or more years of practice (vs 5 or fewer years, aOR 3.75; 95% CI 2.59-5.45), providing medical services to more than 50 persons living with HIV over the past month (vs 50 or fewer persons living with HIV, aOR 3.89; 95% CI 2.83-5.36), and having previously prescribed oPEP (aOR 4.90, 95% CI 3.29-7.29; each *P*<.05). In contrast, unfamiliar with oPEP (aOR 0.12; 95% CI 0.08-0.16), unfamiliar with nPEP (aOR 0.08; 95% CI 0.05-0.11), unaware that risks of UAI exceed percutaneous occupational exposure risk (aOR 0.63; 95% CI 0.42-0.95); self-reported having no written oPEP guideline in place (aOR 0.53; 95% CI 0.35-0.79), and believing that nPEP may promote HIV high-risk behavior (aOR 0.53; 95% CI 0.36-0.77) or result in HIV drug resistance (aOR 0.53; 95% CI 0.36-0.77) among key populations were negatively associated with nPEP prescription behavior (each *P*<.05). However, practicing in provinces with high HIV epidemic level and nPEP pilot programs were not significantly associated with nPEP prescription behavior.

**Table 3:** Univariable and multivariable logistic regression analyses of associations of potential predictors and nPEP prescription history among HIV medical care providers in China (n=777).

Variable				nPEP^a^ prescription					Crude model			Adjusted model			*P* value	
				Yes, n (%)		No (n, %)		OR^b^ (95% CI)			aOR^c^ (95% CI)					
**Age in years**																
	<40			153 (50.2)		152 (49.8)		Ref^d^			—^e^			—		
	40-49			179 (54.1)		152 (45.9)		1.17 (0.86-1.60)			—			—		
	≥50			82 (58.2)		59 (41.8)		1.38 (0.92-2.07)			—			—		
**Ethnicity**																
	Han			374 (52.5)		338 (47.5)		Ref			—			—		
	Non-Han			40 (61.5)		25 (38.5)		1.45 (0.86-2.43)			—			—		
**Sex**																
	Female			233 (55.9)		184 (44.1)		Ref			—			—		
	Male			181 (50.3)		179 (49.7)		0.80 (0.60-1.06)			—			—		
**Educational background**																
	Undergraduate or above			400 (53.8)		343 (46.2)		Ref			—			—		
	Junior college			13 (44.8)		16 (55.2)		0.61 (0.33-1.47)			—			—		
	High school/technical secondary school			1 (20.0)		4 (80.0)		0.21 (0.02-1.93)			—			—		
**Administrative regions of China**																
	North			45 (63.4)		26 (36.6)		Ref			Ref			—		
	Northeast			76 (46.3)		88 (53.7)		0.50 (0.28-0.88)			0.51 (0.28-0.91)			.02		
	East			63 (54.3)		53 (45.7)		0.69 (0.38-1.26)			0.71 (0.38-1.31)			.27		
	South Central			113 (56.2)		88 (43.8)		0.74 (0.43-1.30)			0.82 (0.46-1.45)			.49		
	Southwest			107 (53.8)		92 (46.2)		0.67 (0.39-1.17)			0.70 (0.40-1.23)			.22		
	Northwest			10 (38.5)		16 (61.5)		0.36 (0.14-0.91)			0.35 (0.14-0.89)			.03		
**nPEP pilot province^f^**																
	No			264 (51.8)		246 (48.2)		Ref			Ref			—		
	Yes			150 (56.2)		117 (43.8)		1.20 (0.89-1.61)			1.13 (0.82-1.58)			.46		
**Local HIV epidemic level^g^**																
	Low			60 (51.7)		56 (48.3)		Ref			Ref			—		
	Middle			144 (56.3)		112 (43.8)		1.20 (0.77-1.86)			1.45 (0.85-2.49)			.17		
	High			210 (51.9)		195 (48.1)		1.01 (0.67-1.52)			0.50 (0.24-1.03)			.06		
**Hospital type**																
	General hospital			158 (41.3)		225 (58.7)		Ref			Ref			—		
	Specialized hospital for infectious diseases			256 (65.0)		138 (35.0)		2.64 (1.98-3.53)			2.49 (1.85-3.37)			<.001		
**Technical title**																
	General physician			38 (46.3)		44 (53.7)		Ref			Ref			—		
	Attending physician			141 (51.5)		133 (48.5)		1.23 (0.75-2.01)			1.25 (0.75-2.07)			.40		
	Associate chief physician			105 (47.5)		116 (52.5)		1.05 (0.63-1.74)			0.98 (0.55-1.77)			.96		
	Chief physician			130 (65.0)		70 (35.0)		2.15 (1.28-3.63)			2.16 (1.15-4.05)			.02		
**Clinical practice specialty**																
	Non-HIV care professional			26 (20.3)		102 (79.7)		Ref			Ref			—		
	HIV care professional			388 (59.8)		261 (40.2)		5.83 (3.69-9.22)			6.13 (3.83-9.81)			<.001		
**Practice time in years**																
	≤5			128 (37.1)		217 (62.9)		Ref			Ref			—		
	6-10			118 (64.1)		66 (35.9)		3.03 (2.09-4.40)			3.28 (2.23-4.80)			<.001		
	≥11			168 (67.7)		80 (32.3)		3.56 (2.52-5.02)			3.75 (2.59-5.45)			<.001		
**Unfamiliar with oPEP^h^**																
	No			345 (71.1)		140 (28.9)		Ref			Ref			—		
	Yes			69 (23.6)		223 (76.4)		0.13 (0.09-0.18)			0.12 (0.08-0.16)			<.001		
**Unfamiliar with nPEP**																
	No			264 (85.4)		45 (14.6)		Ref			Ref			—		
	Yes			150 (32.1)		318 (67.9)		0.08 (0.06-0.12)			0.08 (0.05-0.11)			<.001		
**China has issued a national clinical guideline on nPEP**																
	No			115 (50.9)		111 (49.1)		Ref			Ref			—		
	Yes/ I don’t know			299 (54.3)		252 (45.7)		0.04 (0.01-0.11)			0.03 (0.01-0.09)			<.001		
**UAI^i^ risk between males exceeds percutaneous occupational exposure risk**																
	Yes			366 (54.9)		301 (45.1)		Ref			Ref			—		
	No/ I don’t know			48 (43.6)		62 (56.4)		0.64 (0.42-0.96)			0.63 (0.42-0.95)			.03		
**Percutaneous occupational exposure risk exceeds UVI^j^ exposure risk**																
	Yes			238 (44.7)		294 (55.3)		Ref			Ref			—		
	No/I don’t know			176 (71.8)		69 (28.2)		3.15 (2.27-4.37)			3.27 (2.33-4.58)			<.001		
**Number of persons living with HIV in treatment in the past month**																
	≤50			184 (39.9)		277 (60.1)		Ref			Ref			—		
	>50			230 (72.8)		86 (27.2)		4.03 (2.95-5.49)			3.89 (2.83-5.36)			<.001		
**Ever prescribed oPEP**																
	No			264 (45.5)		316 (54.5)		Ref			Ref			—		
	Yes			150 (76.1)		47 (23.9)		3.82 (2.65-5.51)			4.90 (3.29-7.29)			<.001		
**Self-reported having a written oPEP guideline in place**																
	Yes			350 (60.9)		225 (39.1)		Ref			Ref			—		
	No			56 (45.9)		66 (54.1)		0.55 (0.37-0.81)			0.53 (0.35-0.79)			.002		
	Unsure			8 (10.0)		72 (90.0)		0.07 (0.03-0.15)			0.07 (0.03-0.15)			<.001		
**Key populations** **seeking nPEP** **over** **the past 6 months**																
	Never/rarely (<1/month)			149 (32.0)		316 (68.0)		Ref			Ref			—		
	Often/occasionally (>1/month)			265 (84.9)		47 (15.1)		11.96 (8.29-17.25)			13.86 (9.42-20.39)			<.001		
**nPEP-related attitudes**																
	**Adequate time to prescribe nPEP**															
		Disagree	44 (52.4)		40 (47.6)		Ref			Ref			—			
		Neutral	109 (46.4)		126 (53.6)		0.79 (0.48-1.30)			0.83 (0.50-1.38)			.47			
		Agree	261 (57.0)		197 (43.0)		1.20 (0.76-1.92)			1.24 (0.77-2.00)			.38			
	**Feasible to provide nPEP in place**															
		Disagree	3 (37.5)		5 (62.5)		Ref			Ref			—			
		Neutral	11 (19.3)		46 (80.7)		0.40 (0.08-1.93)			0.42 (0.09-2.09)			.29			
		Agree	400 (56.2)		312 (43.8)		2.14 (0.51-9.01)			2.20 (0.51-9.51)			.29			
	**nPEP will promote HIV drug resistance**															
		Disagree	161 (62.6)		96 (37.4)		Ref			Ref			—			
		Neutral	157 (51.0)		151 (49.0)		0.62 (0.44-0.87)			0.65 (0.46-0.92)			.014			
		Agree	96 (45.3)		116 (54.7)		0.49 (0.34-0.72)			0.53 (0.36-0.77)			.001			
	**nPEP will promote HIV risky behavior**															
		Disagree	137 (62.6)		82 (37.4)		Ref			Ref			—			
		Neutral	161 (53.3)		141 (46.7)		0.68 (0.48-0.98)			0.69 (0.48-0.99)			.04			
		Agree	116 (45.3)		140 (54.7)		0.50 (0.34-0.72)			0.53 (0.36-0.77)			.001			
	**Concern about prescribing nPEP**															
		No	13 (54.2)		11 (45.8)		Ref			Ref			—			
		Yes	401 (53.3)		352 (46.7)		0.96 (0.43-2.18)			0.97 (0.43-2.23)			.95			
	**Concern about promoting HIV high-risk behaviors**															
		No	268 (55.1)		218 (44.9)		Ref			Ref			—			
		Yes	146 (50.2)		145 (49.8)		0.82 (0.61-1.10)			0.83 (0.61-1.11)			.21			
	**Concern about poor adherence to nPEP**															
		No	261 (55.9)		206 (44.1)		Ref			Ref			—			
		Yes	153 (49.4)		157 (50.6)		0.77 (0.58-1.03)			0.81 (0.60-1.09)			.16			
	**Concern about HIV drug resistance**															
		No	288 (53.4)		251 (46.6)		Ref			Ref			—			
		Yes	126 (52.9)		112 (47.1)		0.98 (0.72-1.33)			0.97 (0.71-1.32)			.83			
	**Concern about side effects of drugs**															
		No	181 (53.4)		158 (46.6)		Ref			Ref			—			
		Yes	233 (53.2)		205 (46.8)		0.99 (0.75-1.32)			0.98 (0.73-1.31)			.88			
	**Concern about cost of nPEP**															
		No	158 (48.6)		167 (51.4)		Ref			Ref			—			
		Yes	256 (56.6)		196 (43.4)		1.38 (1.04-1.84)			1.39 (1.03-1.86)			.03			
	**Concern about lack of nPEP clinical guideline**															
		No	326 (52.8)		292 (47.2)		Ref			Ref			—			
		Yes	88 (55.3)		71 (44.7)		1.11 (0.78-1.58)			1.19 (0.83-1.71)			.34			
	**Concern about lack of drug indications**															
		No	338 (51.9)		313 (48.1)		Ref			Ref			—			
		Yes	76 (60.3)		50 (39.7)		1.41 (0.95-2.08)			1.50 (1.01-2.24)			.045			
	**Concern about reducing treatment resources of HIV-positive patients**															
		No	377 (57.5)		279 (42.5)		Ref			Ref			—			
		Yes	37 (30.6)		84 (69.4)		0.33 (0.22-0.50)			0.32 (0.21-0.49)			<.001			

^a^nPEP: nonoccupational postexposure prophylaxis.

^b^OR: odds ratio.

^c^aOR: adjusted odds ratio.

^d^Ref: reference.

^e^N/A: not applicable.

^f^nPEP pilot programs were conducted by China CDC in provinces Yunnan, Beijing, Tianjin, Heilongjiang, Hunan, Guangxi, and Guizhou.

^g^Local HIV epidemic level was categorized according to the number of HIV/AIDS cases reported in 2017.

^h^oPEP: occupational postexposure prophylaxis.

^i^UAI: unprotected anal intercourse.

^j^UVI: unprotected vaginal intercourse.

## Discussion

### Principal Findings and Significance

Our study showed that most HIV medical care providers in China were unfamiliar with nPEP, and only a bit more than half of participants had previously prescribed nPEP. We also found that unfamiliarity with nPEP, self-report of having no written PEP-related guideline in place, and less HIV care experience were possibly important barriers to nPEP prescription among HIV medical care providers. This study addresses a gap in the research and shows the negative impact of insufficient knowledge, such as misunderstanding nPEP-related HIV drug resistance and side effects, on the scale-up of nPEP services and subsequent inadequate nPEP prescription by clinicians. It may help public health policymakers learn about HIV medical provider perception of nPEP, thereby providing the opportunity to implement corresponding measures to counter nPEP-related obstacles. Our data also have great significance for further practice after initiating the national nPEP guideline to inform HIV medical care providers in their implementation of nPEP. Additionally, the results of this study are a reference for other countries with similar HIV contexts and insufficient uptake of nPEP services.

### Comparison With Prior Work

We found that the proportion (60.2%) of HIV care providers unfamiliar with nPEP was higher than that reported in a previous study from the United States (51.5%) [19]. About 70% of participants incorrectly thought China had already issued a national clinical guideline on nPEP before this survey. This finding may indicate that a high proportion of HIV medical care providers confused oPEP guidelines, released in 2004 [27], with nPEP guidelines or thought the Chinese Guidelines for Diagnosis and Treatment of HIV/AIDS, updated in 2018 [28], were nPEP guidelines. An accordingly high proportion of prescribing nPEP, though, was not found among these providers. This can be attributed to insufficient familiarity with nPEP because of a lack of media advertisements and tailored training. Newly reported HIV cases in China still show an increasing trend [6], however, with strong acceptance of and great demand for nPEP among key populations [29]. This gap could hinder efforts to curb the spread of the HIV epidemic; therefore, intensified publicity through diverse channels and reinforced training or retraining should be offered to improve the knowledge of these providers.

In our study, the proportion of lifetime prescribing of nPEP among HIV care providers (53.3%) was lower than that reported by previous studies from the United States (67.1%) [23], France (58.0%) [30], and Spain (77.3%) [31], which may indicate a huge gap between China and developed countries in the prevention of HIV spread. The gap between the proportion of HIV medical care providers prescribing nPEP and the demand of key populations for nPEP [29] implies that improving the level of nPEP prescription would likely have a remarkable effect on preventing HIV spread. Previous studies found knowledge plays an indispensable role in PrEP prescription behavior [32,33]. Another study found that HIV-related training has a significant correlation with the increased nPEP and PrEP knowledge and the improved PrEP prescribing practice among HIV care providers [20], which also means a possible effect on nPEP prescription through increasing nPEP knowledge by training. Furthermore, there are many nPEP-related challenges, including risk assessment and management of viral hepatitis, frequent transitions from nPEP to PrEP, and the management of low follow-up rates and poor medication adherence [34], which, if addressed improperly, will bring adverse effects and even harm from nPEP. These challenges will not be resolved in the near-term without targeted nPEP training integrating practical skills exercises into didactic sessions, thereby ultimately delaying the progression of HIV prevention.

### Factors Associated With nPEP Prescription

In addition, we identified independent factors positively correlated with nPEP prescription among HIV medical care providers. Compared with providers working in general hospitals, those in specialized infectious disease hospitals had a significantly higher proportion of prescribing nPEP, probably due to more awareness of HIV-related information. HIV-related stigma remains severe in China, however, and key populations are more inclined to visit the general hospital for HIV-related services to protect their privacy and avoid disclosure [35], which may limit access to nPEP services. Thus, for those providers in general hospitals, reinforced targeted training is necessary to improve their perception and enhance willingness to prescribe nPEP. We also found significantly higher proportions of nPEP prescription among HIV care professionals (vs non-HIV care professionals), chief physicians (vs general physicians), providers with more than 5 years of working experience (vs 5 or fewer years), and those having provided HIV care to more than 50 persons living with HIV over the past month (vs 50 or more persons living with HIV). Providers with professional knowledge, high-ranking technical titles, and rich HIV care experience are usually skilled, which can attract more patients. They as well have more opportunities to attend HIV-related international conferences and obtain information on nPEP from other countries. This finding suggests that nPEP-related training should also be focused on young providers to enrich their nPEP knowledge and improve practical skills, which could even be delivered during student medical training. Moreover, the establishment of specific support mechanisms via senior clinicians would help overcome the obstacles to prescribe nPEP faced by young clinicians.

In contrast, we found that unfamiliarity with nPEP, incorrect beliefs that nPEP will promote HIV drug resistance or high-risk behaviors, self-reported lack of written oPEP guideline in working settings, and unfamiliarity with oPEP were all negatively correlated with nPEP prescription among HIV medical care providers. Although the nPEP guideline was released in October 2020 [26], further outreach efforts to clinicians in working settings are needed or existing incorrect perceptions caused by insufficient nPEP knowledge will continue to impede the scale-up of nPEP services. The oPEP guideline was released about 15 years ago, so HIV medical care providers are more familiar with oPEP (62.4%) than nPEP (39.8%).

The associations between the practice of oPEP and nPEP, two methods targeted at different types of HIV exposure, have rarely been explored in previous studies. In our study, the level of prescribing nPEP was higher among HIV medical care providers who had previously prescribed oPEP than that among those who had not. There are many similar features between nPEP and oPEP about assessing HIV exposure risk, principles of treatment, and the types of antiviral drugs. HIV medical care providers who master the oPEP practice may be relatively more familiar with prescribing nPEP. Therefore, training programs combining nPEP with oPEP can create a synergistic effect on both prescription behaviors of HIV care providers. Notably, despite the emphasis of simplifying prescribing practice from the updated WHO guideline for nPEP [13], regardless of HIV exposure types, different types vary in the risk of acquiring HIV and subsequent laboratory test items [36]. Providers confusing the standards of nPEP and oPEP practices may well prescribe nPEP improperly to some individuals at low risk of HIV acquisition [37] or miss some items, such as pregnancy testing and the collection of forensic specimens [36]. It again underlines the necessity to provide targeted nPEP training or retraining based on the nPEP guideline.

Compared with North China (63.4%), we found a surprisingly lower proportion of nPEP prescription in Northwest (38.5%) and Northeast (46.3%) regions where providers have insufficient nPEP familiarity (30.8% and 33.5%, respectively) and less HIV care practice (23.1% and 32.3%, respectively). In the contrast, the HIV epidemic is highly prevalent in Xinjiang Province, located in Northwest China. Hence, more attention should be paid to these regions, especially the Northwest with its limited resources, in future national nPEP training efforts. Given the difficulty of organizing centralized training for HIV medical care providers from various regions, internet-based online training is critical for nPEP implementation. It has clear advantages for transmitting up-to-date knowledge and ideas, particularly for providers in the Northwest regions with insufficient resources for nPEP implementation. Besides traditional didactic sessions, online simulation trainings related to practical skills are also promising methods to offset the gap of resource from regions.

Finally, there was no significant association between high HIV epidemic level and nPEP prescription behavior of HIV medical care providers. This indicates that key populations in those provinces at high HIV epidemic level may miss the opportunity to obtain nPEP services even after exposure to HIV. Similarly, we did not find the effect of an nPEP pilot program on nPEP prescription behavior of these providers, which may be explained by the relative short duration implementation time and limited number of involved cities. Therefore, it is necessary to further enhance the advertisement of nPEP at a national level to raise the wide attention of HIV medical care providers.

### Strengths and Limitations

Our study has many strengths. First, this is a representative cross-sectional study of nPEP perception and prescribing practice among HIV medical care providers in all 31 provinces of China, and the sources of participants from previous studies have been limited. Second, the sample size of this study was larger than those of previous similar studies. Last, as this is the first study of nPEP perception and prescribing practice among HIV medical care providers in China, the results represent a vital reference that could contribute to solving the obstacles to nPEP prescription, popularizing the use of nPEP nationwide, and controlling HIV spread among key populations.

This study also has limitations. First, this study was conducted by two WeChat groups, and our results rely on self-reporting data, which to some extent would cause sampling bias and reporting bias. Second, HIV care providers from Hong Kong, Macao, and Taiwan were not included in the WeChat groups, and the number of samples from western China (ie, Tibet) was insufficient; hence, the results may not well represent the characteristics of HIV medical care providers from these regions. Additionally, given the cross-sectional design, the causal relationships between prescribing nPEP and other factors are uncertain and will require further prospective studies to confirm.

### Conclusions

This is the first cross-sectional survey of nPEP-related knowledge, attitudes, and prescribing experience among HIV medical care providers in a country without extensive use of nPEP services. Our results underline the insufficient nPEP knowledge and inadequate proportion of nPEP prescription among these providers. Implementing targeted nPEP training or retraining through the internet, particularly for young providers from general hospitals, should be priorities to eliminate obstacles in popularizing nPEP services and ultimately reducing HIV incidence among national key populations.

## Appendix

Multimedia Appendix 1 English version of the questionnaire.

Multimedia Appendix 2 Number of HIV/AIDS cases reported in the 31 provinces of China in 2017.

Multimedia Appendix 3 Checklist for Reporting Results of Internet E-Surveys.

Multimedia Appendix 4 Proportion of nPEP prescriptions in 31 provinces in China.

Multimedia Appendix 5 Forest plot of the results of multivariable logistic regression analysis.

## Abbreviations

aOR: adjusted odds ratio
CHERRIES: Checklist for Reporting Results of Internet E-Surveys
China CDC: Chinese Center for Disease Control and Prevention
IQR: interquartile range
MSM: men who have sex with men
nPEP: nonoccupational postexposure prophylaxis
oPEP: occupational postexposure prophylaxis
OR: odds ratio
PrEP: preexposure prophylaxis
UAI: unprotected anal intercourse
UNAIDS: Joint United Nations Program on HIV/AIDS
UVI: unprotected vaginal intercourse
WHO: World Health Organization
