# Supplementary material for: Context and Barriers to the Prescription of Nonoccupational Postexposure Prophylaxis Among HIV Medical Care Providers: National Internet-Based Observational Study in China
Source: JMIR Public Health Surveill. 2021 Mar 11;7(3):e24234. doi: 10.2196/24234 (PMC7995069; doi:10.2196/24234)
Supplement: Multimedia Appendix 1 [file publichealth_v7i3e24234_app1.docx]

**An online survey on HIV nonoccupational postexposure prophylaxis (nPEP) to HIV medical care providers in China (English version)**

**Part 1. Demographic Characteristics**

**1.1 What department do you work in? [single choice]** *

| ○Infectious Disease Department |
| --- |
| ○HIV/AIDS Department |
| ○Other Departments (Please skip to the end and submit your questionnaire) |

**1.2 Do you provide diagnosis and treatment services for people living with HIV? [single choice]** *

| ○Yes |
| --- |
| ○No (Please skip to the end and submit your questionnaire) |

**1.3 When were you born in: [fill in the blank]** *

_________________________________

**1.4 What is your ethnicity? [single choice]** *

| ○Han |
| --- |
| ○Mongolian |
| ○Manchu |
| ○Hui |
| ○Uyghur |
| ○Others |

**1.5 What is your gender? [single choice]** *

| ○Male |  |  |  |  |  |  |  |
| --- | --- | --- | --- | --- | --- | --- | --- |

○Female

**1.6 What is your education level? [single choice]** *

| ○High school or technical secondary school |
| --- |
| ○Junior college |
| ○Undergraduate or above |

**1.7 Which province are you working in: [fill in the blank]** *

_________________________________

**1.8 What is the administrative level of the hospital you are working in？ [single choice]** *

| ○Provincial level |
| --- |
| ○City level |
| ○County/district level |
| ○Township level |

**1.9 What is the type of the hospital you are working in： [single choice]** *

| ○Specialist hospital for infectious diseases |
| --- |
| ○General hospital |

**1.10 What is your technical title? [single choice]** *

| ○General physician |
| --- |
| ○Attending physician |
| ○Associate chief physician |
| ○Chief physician |
| ○Others |

**1.11 How many years have you practiced in providing HIV/AIDS medical services? (Less than one year shall be counted as one year) [fill in the blank]** *

_________________________________

**1.12 How many HIV/AIDS patients did you treat in the past month? [Fill in the blank]** *

_________________________________

**Part 2. Nonoccupational postexposure prophylaxis (nPEP)**

**2.1 Are you familiar with occupational postexposure prophylaxis (oPEP)? [single choice]** *

| ○Yes |
| --- |
| ○No |

**2.2 Have you prescribed oPEP for people like health care workers or polices? [single choice]** *

| ○Yes |
| --- |
| ○No |

**2.3 Are you familiar with nonoccupational postexposure prophylaxis (nPEP)? [single choice]** *

| ○Extremely familiar |
| --- |
| ○Very familiar |
| ○Generally familiar |
| ○Not familiar very much |
| ○Not familiar at all |

**2.4 Do you think China has issued a national clinical guideline on nPEP?** *

| ○Yes |
| --- |
| ○No |
| ○I don’t know |

**2.5 Do you think unprotected anal intercourse (UAI) risk exceeds percutaneous occupational exposure risk? [single choice]** *

| ○Yes |
| --- |
| ○No |
| ○I don’t know |

**2.6 Do you think percutaneous occupational exposure risk exceeds unprotected vaginal intercourse (UVI) exposure risk? [single choice]** *

| ○Yes |
| --- |
| ○No |
| ○I don’t know |

**2.7 Have you prescribed nPEP for people with HIV risks? [single choice]** *

| ○Yes |
| --- |
| ○No |

**2.8 Is there a written oPEP guideline in your working place?** *

| ○Yes |
| --- |
| ○No |

**2.9 How often do you encounter key populations seeking nPEP help over the past 6 months? [single choice]** *

| ○Often (more than 1 per week) |
| --- |
| ○Occasionally (more than 1 per month) |
| ○Rarely (less than 1 per month) |
| ○Never |

**2.10 Are there any barriers for prescribing nPEP in your working place?** *

| ○Yes |
| --- |
| ○No |

**2.11 Do you agree that prescribing nPEP in clinical settings is feasible? [single choice]** *

| ○Agree |
| --- |
| ○Neutral |
| ○Disagree |

**2.12 Do you agree that clinicians have enough time to prescribe nPEP? [single choice]** *

| ○Agree |
| --- |
| ○Neutral |
| ○Disagree |

**2.13 Do you agree that prescribing nPEP will promote HIV drug resistance? [single choice]** *

| ○Agree |
| --- |
| ○Neutral |
| ○Disagree |

**2.14 Do you agree that prescribing nPEP will promote high-risk behaviors? [single choice]** *

| ○Agree |
| --- |
| ○Neutral |
| ○Disagree |

**2.15 Are you worry about being blamed for prescribing nPEP due to no nPEP drug indication? [single choice]** *

| ○Yes |
| --- |
| ○No |

**2.16 Would you recommend nonoccupational postexposure prophylaxis (nPEP) for following people? [Matrix multiple choice]** *

|  | Never recommend | Rarely recommend | Often recommend | Extremely recommend | I don’t know |
| --- | --- | --- | --- | --- | --- |
| people having a partner living with HIV | □ | □ | □ | □ | □ |
| people with HIV status-unknown partners | □ | □ | □ | □ | □ |
| people who had been sexually assaulted | □ | □ | □ | □ | □ |
| people with histories of unprotected sexual intercourse | □ | □ | □ | □ | □ |
| people with histories of sexually transmitted disease | □ | □ | □ | □ | □ |
| people with histories of drug injection | □ | □ | □ | □ | □ |
| people with histories of irregular visits to the clinic | □ | □ | □ | □ | □ |
| people with histories of poor drug adherence | □ | □ | □ | □ | □ |

**2.17 What do you think are the main problems of prescribing nPEP in China? [multiple choice]** *

| □Increased risk behavior |
| --- |
| □Poor medication adherence |
| □HIV drug resistance |
| □Side effects |
| □High cost |
| □No specific guidance for nPEP |
| □No nPEP drug indication |
| □Resources reduced for HIV-positive patients |
| □Other problems |
| □No problems |

**2.18 Is it necessary to have expert consensus for nPEP [single choice]** *

| ○Yes |
| --- |
| ○No |

**2.19 Is it necessary to establish outpatient for nPEP? [single choice]** *

| ○Yes |
| --- |
| ○No |

**The questionnaire is over. Thank you for your participation.**
