# Supplementary material for: Context and Barriers to the Prescription of Nonoccupational Postexposure Prophylaxis Among HIV Medical Care Providers: National Internet-Based Observational Study in China
Source: JMIR Public Health Surveill. 2021 Mar 11;7(3):e24234. doi: 10.2196/24234 (PMC7995069; doi:10.2196/24234)
Supplement: Multimedia Appendix 5 [file publichealth_v7i3e24234_app5.doc]

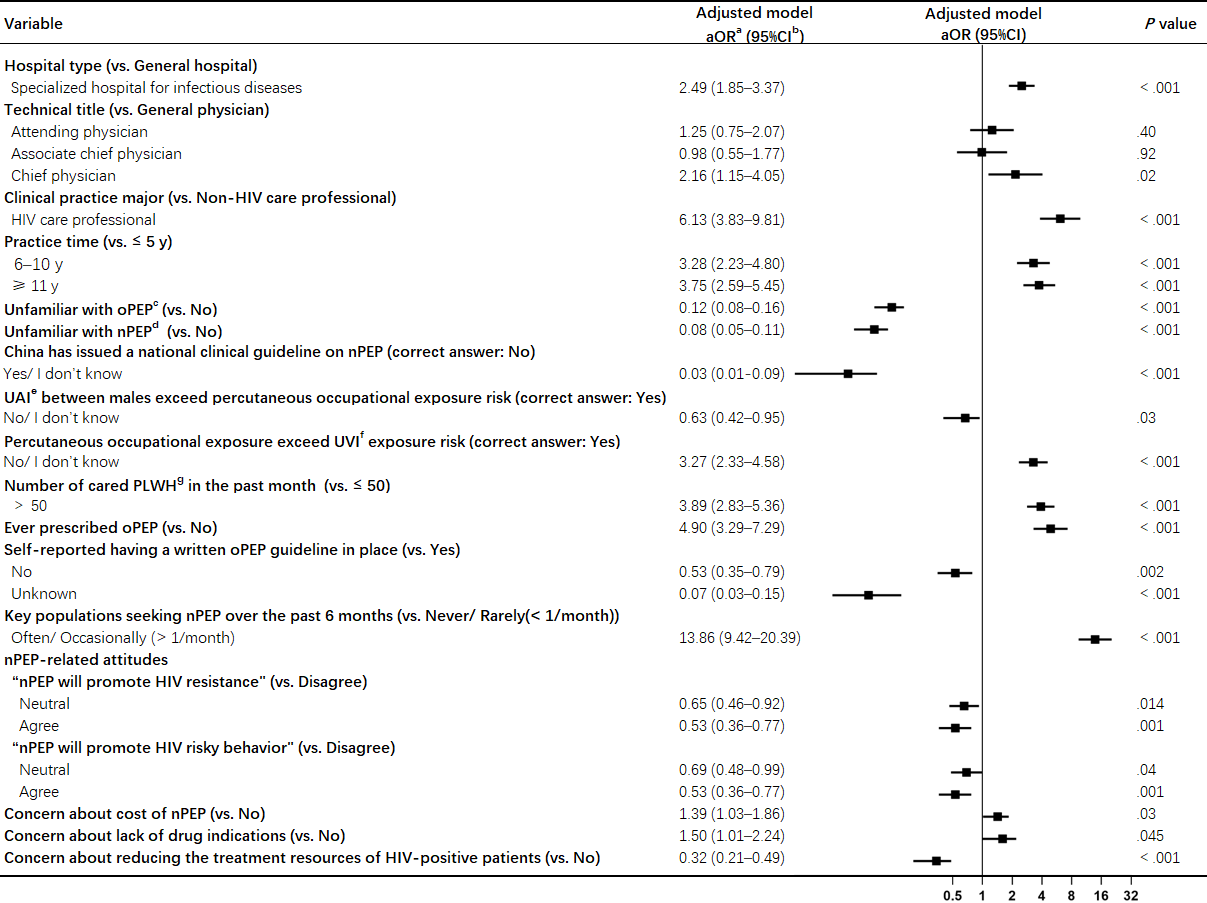


Figure. Multivariable logistic regression analysis to identify predictors of nPEP prescription history among HIV medical care providers

Adjusted covariates: age, sex, ethnicity, educational background, and administrative regions.

^a^aOR, adjusted odds ratio.

^b^CI, confidence interval.

^c^oPEP, occupational postexposure prophylaxis.

^d^nPEP, nonoccupational postexposure prophylaxis.

^e^UAI, unprotected anal intercourse.

^f^UVI, unprotected vaginal intercourse.

^g^PLWH, persons living with HIV.
